# Patient outcome prognosis for external beam radiation therapy using CBCT-based radiomics: a systematic review

**DOI:** 10.1088/2057-1976/ae308b

**Published:** 2026-01-06

**Authors:** Chih-Wei Chang, Tonghe Wang, Richard L J Qiu, Xiang Li, Jochen Cammin, Kailin Yang, Yue-Houng Hu, Lei Ren, Ping Xia, Amit Sawant, Jacob Scott, Jeffrey Buchsbaum, Xiaofeng Yang

**Affiliations:** 1 Department of Radiation Oncology and Winship Cancer Institute, Emory University, Atlanta, GA, United States of America; 2 Department of Medical Physics, Memorial Sloan Kettering Cancer Center, New York City, NY, United States of America; 3 Department of Radiation Oncology, University of Maryland, Baltimore, MD, United States of America; 4 Department of Radiation Oncology, Holden Comprehensive Cancer Center, Iowa Neuroscience Institute, University of Iowa, Iowa City, IA, United States of America; 5 Department of Radiation Oncology, Harvard University, Boston, MA, United States of America; 6 Department of Radiation Oncology, Cleveland Clinic Foundation, Cleveland, OH, United States of America; 7 National Cancer Institute, Division of Cancer Treatment and Diagnosis, National Cancer Institute, Bethesda, MD, United States of America

**Keywords:** CBCT, radiomics, radiotherapy, outcome prediction

## Abstract

*Objective*. This review investigates the use of cone-beam computed tomography (CBCT) in conjunction with radiomics for external beam radiation therapy (EBRT) in cancer treatment. CBCT, which provides high-resolution, volumetric images, offers a promising tool for precision treatment delivery. By integrating radiomics and quantitative features extracted from CBCT, this review explores potential advancements in tumor characterization, treatment planning, and monitoring treatment responses in personalized cancer therapy. *Approach*. We conducted this systematic review using the PRISMA (preferred reporting items for systematic reviews and meta-analyses) framework. This study focused on CBCT-only radiomics applications, examining publications in PubMed, Embase, and Scopus databases. The inclusion criteria were strictly peer-reviewed journal articles, resulting in 29 studies being selected for analysis. These studies were divided into two main categories: (1) method development for treatment outcome prediction; (2) verification, validation, and uncertainty quantification (VVUQ) for CBCT-based radiomics. *Main Results*. The literature encompasses a range of investigations into CBCT-based radiomics for EBRT, covering different cancer types such as head-and-neck squamous cell carcinoma, non-small cell lung cancer, esophageal squamous cell cancer, hepatocellular carcinoma, prostate cancer, and rectal cancer. These studies used radiomics to predict outcomes including tumor response, local failure, tissue toxicity, and patient survival. VVUQ studies addressed the robustness and reproducibility of radiomic features. Furthermore, the emerging field of 4D-CBCT radiomics shows potential in improving image quality. *Significance*. CBCT-based radiomics presents a promising advancement in personalized radiotherapy, allowing for enhanced cancer prognosis and treatment adaptation. However, challenges of imaging quality and acquisition need to be addressed to ensure consistency and reliability. Future research should focus on standardizing imaging protocols and incorporating multi-institutional collaborations to further validate the clinical applicability of CBCT-based radiomics. Integration of this technology can potentially induce a paradigm shift in personalized cancer radiotherapy. New technologies promise to make CBCT even more valuable in the future.

## 1. Introduction

The field of cancer radiotherapy has undergone significant evolution over recent decades, with advancements in imaging technologies (Pereira *et al* 2014, Jiang and Thomas 2021) playing a pivotal role in enhancing treatment precision and outcomes. Cone-beam computed tomography (CBCT) has emerged as one of the fundamental daily imaging modalities (Srinivasan *et al* 2014) in radiation oncology, offering high-resolution, volumetric images that facilitate improved tumor targeting and treatment verification (Chang *et al* 2023). Concurrently, radiomics (van Timmeren *et al* 2020, Tourassi 1999, Castellano *et al* 2004, Mannil *et al* 2018, Neisius *et al* 2019), a field involving the extraction and analysis of large quantities of quantitative features from medical images, has emerged as a promising approach to personalize and optimize cancer treatment. This review aims to explore the perspectives on utilizing CBCT for radiomics in cancer radiotherapy, discussing its potential benefits, inherent challenges, and future directions.

The genesis of image-guided radiotherapy can be traced back to the use of two-dimensional (2D) x-rays (Connell and Hellman 2009), which provided rudimentary anatomical information for treatment planning. The limitations of these early techniques in accurately targeting tumors and sparing healthy tissues precipitated the development of three-dimensional (3D) imaging technologies. Contemporary image-guided external beam radiation therapy (EBRT) can incorporate daily CBCT scans to ensure precise irradiation of the target volume. CBCT, initially developed for dental and maxillofacial imaging in 2001 (Miracle and Mukherji 2009, Kaasalainen *et al* 2021), was rapidly adopted in radiation oncology in 2002 (Jaffray *et al* 2002) due to its capacity to provide in-room, high-resolution, volumetric images at the time of treatment. CBCT systems have been integrated into linear accelerators, enabling image-guided radiation therapy (IGRT) and enhancing the accuracy of radiation delivery for EBRT.

Radiomics (Gardin *et al* 2019, Lohmann *et al* 2020, Mayerhoefer *et al* 2020), introduced in the early 2000s, has garnered significant attention for its potential to provide non-invasive biomarkers derived from medical imaging. Through the extraction and analysis of large sets of quantitative features (Traverso *et al* 2018), radiomics can reveal patterns imperceptible to the human eye, thereby facilitating personalized treatment strategies. The advancement of computer hardware and machine learning algorithms in radiomics have precipitated a paradigm shift in medical image analysis from qualitative analysis to quantitative prognosis (Rogers *et al* 2020). The contemporary EBRT workflow incorporates daily CBCT scans to minimize positional uncertainty, ensuring treatment quality. These daily images potentially offer a means of forecasting early tumor response or normal tissue toxicity when integrated with radiomics techniques (Zhang *et al* 2017, Desideri *et al* 2020). However, CBCT is susceptible to various artifacts (Schulze *et al* 2011), including scatter, beam hardening, and motion artifacts. These artifacts can degrade image quality and introduce inherent uncertainties that may bias radiomic analyses.

Ismail *et al* (2024) reviewed CBCT-based radiomics studies in head and neck cancers, revealing that the current literature remains extremely limited, with only a handful of studies meeting inclusion criteria. The existing studies were predominantly retrospective, single-institutional designs with modest quality scores, lacking phantom validation studies and prospective trials. Key challenges identified include unstandardized image acquisition protocols and batch effects that can compromise radiomics feature reproducibility and model performance. Despite these limitations, the included CBCT-based studies demonstrated potential applications in detecting tumor volume changes during treatment, predicting local failure, and assessing treatment response. However, this review was restricted to head and neck cancers and did not comprehensively address methodological rigor across broader radiation oncology applications. Building upon this foundation, our work aims to systematically review the current status of CBCT utilization for radiomics across radiation oncology, with a specific focus on method development and verification, validation, and uncertainty quantification (VVUQ).

## 2. Materials and methods

This study adopted the preferred reporting items for systematic reviews and meta-analyses (PRISMA) protocol (Page *et al* 2021a, 2021b) to systematically and transparently evaluate the current state of CBCT-enabled radiomics in EBRT, with a focus on tumor prognosis, normal tissue toxicity, and patient survival outcomes. PRISMA 2020 represents a comprehensive reporting guideline designed to enhance transparency and completeness in systematic review reporting, consisting of a checklist covering all aspects from title to data availability. Originally published in 2009 and updated in 2020, PRISMA was developed through a rigorous multi-stage process involving literature review of 60 reporting guidance documents, a survey of 110 systematic review methodologists and journal editors, and a two-day international consensus meeting with 21 experts. The guideline emphasizes complete reporting of methods and results to enable readers to assess the appropriateness of review methods, evaluate the trustworthiness of findings, and facilitate replication and updating of reviews while reducing research waste.

The investigation specifically emphasized CBCT-only radiomics applications, as this imaging modality is routinely acquired prior to each daily treatment session, and these patient-specific daily images are hypothesized to contain a substantial amount of latent information pertinent to treatment outcomes. Following PRISMA 2020 recommendations, this systematic review employed explicit and reproducible methods for literature identification, study selection, data extraction, and synthesis to ensure methodological rigor and transparency. The primary objectives of this investigation were to elucidate the limitations and challenges associated with current CBCT-based radiomics and to delineate future perspectives in this field. To ensure the credibility and rigor of this review, only peer-reviewed journal publications were included in the analysis, thereby focusing on formally validated and quality-controlled research contributions to the field.

### 2.1. Search strategies

**Figure 1.**
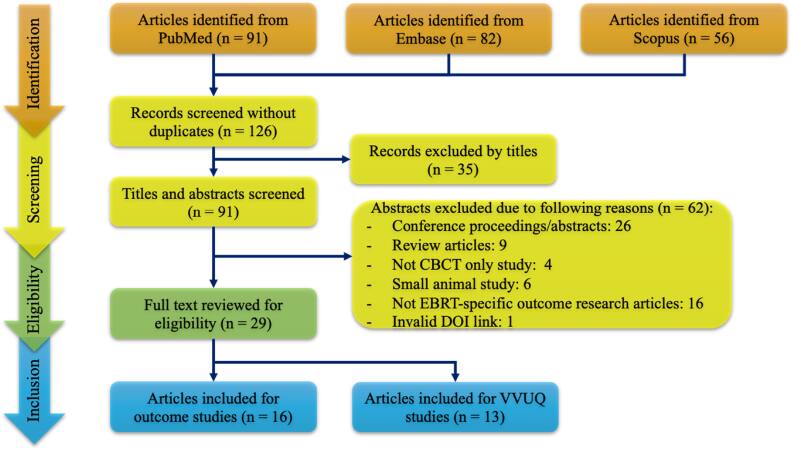
Meta-analysis flow diagram illustrating the reference searching strategy from PRISMA statement for identifying articles regarding CBCT-based radiomics for EBRT. The literature search includes databases of PubMed, Embase, and Scopus. After screening using the PRISMA strategy, a total of 29 studies were included in this systematic review work.

The literature survey was conducted utilizing three primary databases: PubMed (n = 91), Embase (n = 82), and Scopus (n = 56). The search encompassed all available documents from the inception of each database through October 2025. To maximize the initial search domain, a parsimonious search criterion was employed, utilizing the terms ‘CBCT’ and ‘radiomics’ in combination. Figure 1 illustrates the initial search results. Following the removal of duplicate entries, 126 studies were subjected to a title review. This process resulted in the exclusion of multi-modality investigations, leaving 91 articles for further consideration. Subsequently, an abstract review was conducted, leading to the exclusion of an additional 62 studies based on the following criteria: conference proceedings or abstracts, review articles, non-CBCT-only investigations, small animal studies, non-EBRT-related research or invalid DOI link. The final corpus for this review comprises 29 articles, which can be further stratified into two primary categories: method development for treatment outcome prediction (n = 16) and VVUQ for CBCT-based radiomics (n = 13).

## 3. Results

### 3.1. Statistics of literature search results

**Figure 2.**
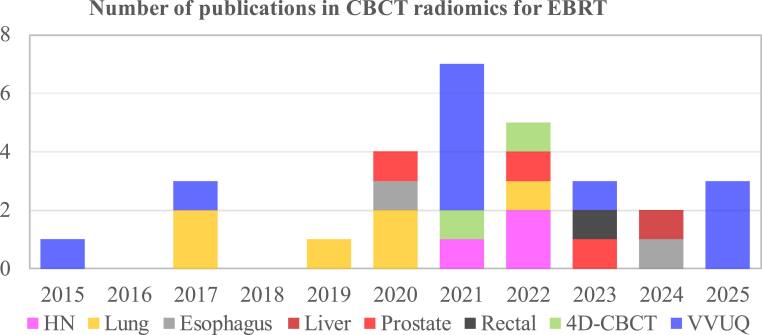
Overview of peer-reviewed research articles in CBCT-based radiomics for EBRT. The VVUQ category includes the publications contributing to the reproducibility and stability of CBCT-derived radiomics features. The papers published in 2025 include only those from the first ten months.

The temporal trend of peer-reviewed publications in CBCT-based radiomics for EBRT is illustrated in figure 2. Commencing in 2015, the literature encompasses 29 articles with 16 studies focused on developing radiomics models for treatment outcomes and cancer prognosis, and 13 studies focused on VVUQ of radiomics methods. The method development investigations were categorized according to different cancer diagnosis including 3 studies (Morgan *et al* 2021, Iliadou *et al* 2022, Sellami *et al* 2022) on head-and-neck squamous cell carcinoma (HNSCC), 6 (van Timmeren *et al* 2017a, 2017b, 2019, Qin *et al* 2020, Shi *et al* 2020, Das *et al* 2022) on non-small cell lung cancer (NSCLC), 2 (Du *et al* 2020, Nakamoto *et al* 2024) on esophageal squamous cell cancer (ESCC), 1 (Yang *et al* 2024) on hepatocellular carcinoma, 3 (Bosetti *et al* 2020, Delgadillo *et al* 2022, Mendes *et al* 2023) on prostate cancer, and 1 (Li *et al* 2023) on rectal cancer. The VVUQ work included 2 studies using 4D-CBCT.

### 3.2. CBCT-based radiomics according to cancer diagnosis

**Figure 3.**
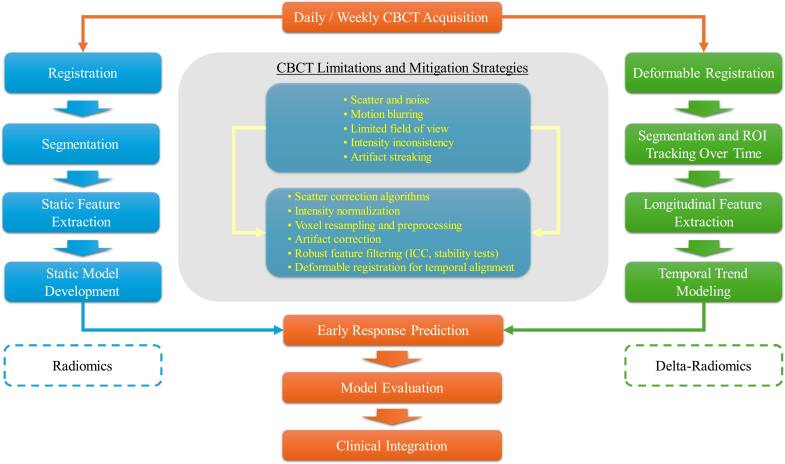
Workflow illustrating how daily or weekly CBCT images are transformed into quantitative biomarkers for treatment response assessment. The left pathway represents the static radiomics pipeline, and the right pathway illustrates the delta radiomics workflow, where deformable registration enables temporal tracking of regions of interest and extraction of longitudinal features for trend analysis. A central panel summarizes common CBCT limitations, along with mitigation strategies. Both static and temporal radiomics pipelines contribute to early response prediction, model evaluation, and eventual clinical integration.

Figure 3 presents an overview of the standard workflow used for CBCT-based radiomics and delta radiomics in radiotherapy, summarizing how the reviewed studies transform routine image guidance data into quantitative biomarkers. The process typically begins with daily or weekly CBCT acquisition during treatment, followed by a series of preprocessing steps designed to address modality-specific limitations such as scatter-related noise, restricted field of view, motion blurring, artifact streaking, and inconsistent voxel intensities. These steps often include voxel resampling, gray level normalization, and scatter or artifact correction, which help stabilize the images for quantitative analysis. Registration and segmentation are then performed to ensure spatial correspondence across fractions and to accurately delineate regions of interest. Radiomic features are extracted from original and transformed image sets, after which multiple layers of feature robustness testing are applied, including reproducibility filtering, correlation analysis, and statistical or model-based reduction. Delta-radiomics extends this framework by analyzing temporal trajectories of features obtained from serial CBCT scans, allowing the quantification of treatment-induced changes in tumor and normal tissue over time. Longitudinal modeling techniques, such as least squares trend fitting or time-dependent feature analysis, are used to capture biologically meaningful patterns that may not be evident in single-time point imaging. The resulting feature subsets are incorporated into machine learning or statistical models for outcome prediction, including treatment response, toxicity risk, or prognostic stratification. Together, these steps establish CBCT radiomics and delta radiomics as practical tools for extracting dynamic biological information during radiotherapy, enabling early response assessment, adaptive treatment decision making, and personalized patient management despite the inherent limitations of CBCT imaging.

Table 1 provides a comprehensive summary of the CBCT-based radiomics investigations across various cancer types. Two investigations (Qin *et al* 2020, Yang *et al* 2024) utilized CBCT images derived from stereotactic body radiation therapy (SBRT), while three studies (van Timmeren *et al* 2017a, Iliadou *et al* 2022, Yang *et al* 2024) employed delta-radiomics features. The objectives of these human studies encompassed a range of predictive outcomes, including local failure, tumor response, patient survival, and tissue toxicity. The quantity of initial radiomic feature extraction exhibited considerable variation, ranging from a minimum of 31 features (Bosetti *et al* 2020) to a maximum of 1131 features (Nakamoto *et al* 2024). This distribution of studies across cancer types and the diversity in both methodological approaches and feature selection underscored the burgeoning interest in CBCT-based radiomics across multiple radiation oncological domains. The variability in feature selection also highlighted the ongoing challenge of identifying optimal radiomics signatures for specific clinical endpoints.

**Table 1.** Summary of CBCT-based radiomics studies with different treatment characteristics and different objectives of using machine learning models.

References	Diagnosis	Prescribed dose (Gy)	Initial radiomics feature extraction	Feature Selection Method	Model	Prediction
Morgan *et al* (2021)	HNSCC	70	102	ICC ≥ 0.95, mRMR, RFECV	EBM	Local failure
Iliadou *et al* (2022)^b^	HNSCC	66	104	RFE with correlation bias	SVM	Anatomical changes
Sellami *et al* (2022)	HNSCC	70	88	Longitudinal stability filtering	Logistic regression	Cancer prognosis
van Timmeren *et al* (2017a)^b^	NSCLC	45–73.8	232	Longitudinal change filtering	smallest detection	Early tumor response
van Timmeren *et al* (2017b)	NSCLC	≥ 40	1119	Multivariable model-based reduction	Cox/Linear regression	Survival
van Timmeren *et al* (2019)	NSCLC	45–76	283	Trend-based filtering	Cox regression	Survival
Qin *et al* (2020)^a^	NSCLC	—	187	Correlation filtering	Multivariate logistic regression	Lung toxicity, cancer prognosis
Shi *et al* (2020)	NSCLC	≥ 58	658	Test–retest robustness filtering	Linear regression	Survival
Das *et al* (2022)	NSCLC	60	107	Triage-learning internal selection	Neural network	Early tumor response
Du *et al* (2020)	ESCC	≥ 50	851	ICC, multivariate reduction	Logistic LASSO regression	Lung toxicity
Nakamoto *et al* (2024)^b^	ESCC	50.4	1131	Univariate significance testing	Regularized Cox regression	Survival
Yang *et al* (2024)^a,b^	Hepatocellular carcinoma	45, 48, 50	547	ICC, volume-correlation filtering	Logistic regression	Tumor response
Bosetti *et al* (2020)	Prostate cancer	78	31	Reproducibility filtering, model-driven reduction	Logistic regression	Biochemical relapse
Delgadillo *et al* (2022)^b^	Prostate cancer	70.2–91.2	42	Lloyd-Max quantization	Logistic regression	Genitourinary toxicity
Mendes *et al* (2023)	Prostate cancer	—	107	ANOVA K-best, RFE with SVR, RFECV	Neural network	Distinguishing favorable and unfavorable outcomes
Li *et al* (2023)	Rectal cancer	50.4	93	Z-score normalization, outlier removal, U-test filtering	Logistic regression	Tumor response

*Abbreviations:* HNSCC = head-and-neck squamous cell carcinoma; NSCLC = non-small cell lung cancer; ESCC = esophageal squamous cell cancer; EBM = explainable boosting machine; SVM = support vector machine; SVR = support vector regression; LASSO = least absolute shrinkage and selection operator; ICC = intra-class correlation coefficient; mRMR = maximum relevance minimum redundancy; RFECV = recursive feature elimination with cross-validation; RFE = recursive feature elimination with correlation bias. ANOVA = analysis of variance.

^a^ Stereotactic body radiation therapy (SBRT).

^b^ Including delta-radiomics features.

Table 2 provides a comprehensive summary of the data quality metrics for each human study included in this review. The sample sizes exhibited considerable variation, ranging from a minimum of 23 (Shi *et al* 2020) to a maximum of 141 (van Timmeren *et al* 2019) participants. Notably, two studies utilized multi-institutional datasets, potentially enhancing the generalizability of their findings. The CBCT images analyzed in these studies were acquired using linear accelerators (LINACs) from two major vendors: Varian Medical Systems (Palo Alto, USA) and Elekta AB (Stockholm, Sweden). The x-ray tube voltages employed in image acquisition ranged from 100 to 140 kVp, reflecting the diversity in imaging protocols across institutions and equipment manufacturers. This heterogeneity in sample sizes, data sources, and imaging parameters emphasized the importance of considering these factors when interpreting and comparing results across studies. The inclusion of multi-institutional datasets in some studies (van Timmeren *et al* 2017b, 2019) represented a positive trend towards more robust and generalizable findings in the field of CBCT-based radiomics for external beam radiation therapy.

**Table 2.** Summary of data quality for each CBCT-based radiomics study.

References	Years of data collection	Institution	No. of patients	Primary set made public	Vendor	X-ray tube voltage (kVp)
Morgan *et al* (2021)	2014–2019	Single	90	No	Varian	100, 125
Iliadou *et al* (2022)	—	Single	40	No	—	—
Sellami *et al* (2022)	2014–2017	Single	93	No	Varian	100
Timmeren *et al* (van Timmeren *et al* (2017a)	2013–2015	Single	115	No	Varian	125
Timmeren *et al* (van Timmeren *et al* (2017b)	2012–2014, 2009–2011, 2007–2011	Multiple (n = 3)	132, 62, 94	Yes,		
				No,		
				No	Varian, Elekta, Elekta	125, 120, 120
Timmeren *et al* (van Timmeren *et al* (2019)	2012–2015, 2007–2011, 2010–2016, 2009–2011	Multiple (n = 4)	141, 94, 61, 41	No	Varian, Elekta, Varian, Elekta	125, 120, 120, 120
Qin *et al* (2020)	2016–2018	Single	34	No	Varian	110
Shi *et al* (2020)	2011–2016	Single	23	No	Elekta	120
Das *et al* (2022)	—	Single	99	No	Varian	80
Du *et al* (2020)	2017–2019	Single	96	No	Varian	125
Nakamoto *et al* (2024)	2010–2016	Single	26	No	Elekta	120
Yang *et al* (2024)	2015–2019	Single	49	No	Varian	110, 125
Bosetti *et al* (2020)	2009–2013	Single	31	No	Varian	125
Delgadillo and Spieler *et al* (Delgadillo *et al* (2022)	—	Single	50	No	Varian	125
Mendes *et al* (2023)	2019–2021	Single	70	No	Varian	125, 140
Li *et al* (2023)	—	Single	30	No	—	—

#### 3.2.1. Head-and-neck cancers

Across the three studies summarized in table 1, CBCT-based radiomics and machine learning have been explored as valuable tools for enhancing head and neck radiotherapy by capturing treatment-related anatomical and textural changes that are not readily visible through standard clinical assessment. Although CBCT imaging is known to have important limitations, including restricted field of view (FOV) and reduced image quality due to scatter, noise, and intensity inconsistencies, each study applied specific strategies to manage these challenges and extract reliable radiomic information. Morgan *et al* (2021) reduced the impact of CBCT image quality constraints by integrating CBCT radiomic features with higher quality CT-based radiomics and clinical variables within an ensemble predictive model. By combining modalities with complementary strengths, they demonstrated improved prediction of local failure and illustrated how CBCT can contribute a meaningful signal when used alongside planning CT. Iliadou *et al* (2022) addressed CBCT limitations through deformable registration, careful preprocessing, and the use of delta-radiomics derived from weekly CBCT scans. This delta-based approach relies on relative changes from a consistent baseline rather than absolute intensity values, which helps reduce sensitivity to CBCT acquisition variability and field of view restrictions. Sellami *et al* (2022) applied a rigorous workflow aligned with radiomic standards of image biomarker standardization initiative (IBSI) and incorporated a longitudinal feature selection strategy to identify only those CBCT-derived features that remained stable and reproducible across treatment. Their results showed that even within the constraints of CBCT imaging, robust temporal radiomic patterns can forecast treatment response at early stages. Collectively, these studies show that although CBCT is not optimized for quantitative radiomics, careful methodological design, including multimodal integration, deformable alignment, delta-based normalization, and stability-based feature filtering, can substantially mitigate CBCT limitations. The combined evidence highlights a clear direction for the field, in which longitudinal CBCT radiomics provides an emerging opportunity to improve early prediction of treatment response, guide adaptive radiotherapy decisions, and identify patients at elevated risk of local failure, ultimately supporting more personalized and proactive head and neck cancer management.

#### 3.2.2. Lung cancers

The six studies included in table 1 demonstrate that CBCT-based radiomics has increasingly been investigated as a means of enhancing treatment evaluation for NSCLC. Although CBCT suffers from fundamental physical limitations, including increased scatter, reduced soft tissue contrast, voxel intensity instability, and a restricted FOV, each study implemented methodological strategies to mitigate these challenges and extract clinically meaningful image biomarkers. The early work by van Timmeren *et al* (2017b) demonstrated that stable radiomic features can be selected from longitudinal CBCT acquisitions and that temporal changes in these features, known as delta radiomics, capture biologically relevant treatment-induced alterations that are not apparent in static imaging. Their subsequent investigations (van Timmeren *et al* 2017b, 2019) further showed that longitudinal CBCT radiomic signatures provide prognostic information for overall survival and locoregional recurrence that complements baseline CT features, highlighting the added value of intra-treatment imaging. Qin *et al* (2020) incorporated Monte Carlo-based scatter corrected CBCT images and demonstrated that the combination of CBCT and pretreatment CT features improved the prediction of lung toxicity compared to CT-based features alone. Shi *et al* (2020) applied delta radiomics to characterize treatment response in locally advanced disease, reinforcing the feasibility of serial CBCT as a response monitoring tool. Das *et al* (2022) extended the clinical scope of CBCT radiomics by showing that CBCT-derived features can stratify early responders and non-responders during conventionally fractionated radiotherapy using a triage learning framework. Taken together, these studies form a coherent body of evidence demonstrating that, when supported by appropriate correction, normalization, and stability filtering, CBCT radiomics can yield robust quantitative biomarkers despite the modality’s inherent imaging constraints.

Across these studies, the findings from these six investigations indicate a clear evolution in the role of CBCT from a device primarily used for geometric verification to an imaging modality capable of providing dynamic biological information throughout the course of radiotherapy. By leveraging methodological safeguards such as scatter correction, delta-based normalization, deformable alignment, feature stability filtering, multimodal integration with planning CT, and machine learning models resilient to noisy inputs, researchers have demonstrated that longitudinal radiomic signatures can be extracted from CBCT with sufficient fidelity to support clinically relevant predictions. The consistent observation that radiomic trajectories outperform or augment static pretreatment features suggests that intra-fractional tumor behavior, rather than baseline morphology alone, may hold additional prognostic and therapeutic significance. Moreover, these studies illustrate the potential of CBCT radiomics to inform early adaptive radiotherapy, either by identifying patients likely to benefit from plan modification or by detecting suboptimal response trajectories before they manifest on conventional follow-up imaging. The incorporation of CBCT radiomics into toxicity prediction frameworks further expands its clinical utility, indicating that daily or weekly changes in anatomical and textural features may reflect subtle interactions between dose delivery, lung parenchymal response, and patient-specific radiosensitivity. As CBCT acquisition and reconstruction techniques continue to improve, and as harmonization strategies mature, these findings point toward a future in which CBCT functions as a routinely available, cost-effective, and information-rich imaging source for monitoring tumor and normal tissue dynamics. Such developments hold promises for more personalized, adaptive, and biologically guided radiotherapy strategies in NSCLC, aligning with the broader shift toward data-driven precision oncology.

#### 3.2.3. Esophagus cancers

The two studies summarized in table 1 illustrate how CBCT-based radiomics is beginning to shape toxicity prediction and treatment response assessment in esophageal cancer, despite the modality’s known limitations. Du *et al* (2020) addressed these challenges by applying standardized CBCT acquisition protocols, semiautomatic lung segmentation, and a multistage feature selection process that removed unstable or highly correlated features before constructing a radiomics-informed nomogram for predicting radiation pneumonitis. Their findings showed that early-treatment CBCT features, when combined with dosimetric factors such as V5 and mean lung dose, substantially improved risk stratification compared to clinical or dosimetric parameters alone, demonstrating that meaningful quantitative information can be recovered from CBCT when appropriate preprocessing and normalization steps are applied. Building on this direction, Nakamoto *et al* (2024) explored delta-radiomics derived from serial CBCT images in patients undergoing chemoradiotherapy for esophageal squamous cell carcinoma and identified temporal textural changes associated with treatment sensitivity and prognosis. By focusing on radiomic trajectories rather than single-time-point measurements, their work highlights the potential of CBCT as a low-cost, intra-treatment biomarker source capable of capturing evolving tumor and tissue response patterns that conventional imaging or clinical assessment may miss. From a broader perspective, these investigations reflect an emerging narrative in which CBCT, once used solely for geometric verification, is increasingly leveraged as a quantitative imaging tool. With carefully designed correction methods, stability-oriented feature selection, and longitudinal modeling, CBCT radiomics is emerging as a feasible approach for early toxicity prediction and biologically informed treatment adaptation in esophageal cancer.

#### 3.2.4. Liver cancers

Yang *et al* (2024) investigated the feasibility of using original and delta CBCT radiomics to predict treatment response in liver tumors undergoing SBRT, as summarized in table 1, providing an early example of how intra-treatment CBCT can function as a quantitative biomarker despite well-known CBCT quality limitations. To manage these challenges, the authors implemented a rigorous preprocessing strategy that included deformable registration of planning CT (pCT) to each CBCT fraction, manual verification of target volumes, voxel resampling to a uniform grid, and gray-level normalization to reduce variability introduced by CBCT acquisition parameters. They also applied a multi-step feature screening pipeline incorporating robustness analysis via intraclass correlation coefficients, correlation filtering against tumor volume, and assessment of CBCT-pCT feature interchangeability, ensuring that only stable and reproducible radiomic features were retained for modeling. By analyzing CBCT features across treatment fractions, the study demonstrated that delta radiomics captured dynamic tumor changes more effectively than single-time-point features, with combined original and delta features yielding the highest predictive accuracy for distinguishing local efficacy categories in a leave-one-out cross-validation framework. This work illustrates an emerging narrative in which daily CBCT, traditionally used for setup verification, can be transformed into a source of biologically informative longitudinal imaging. Through careful normalization, robustness testing, and longitudinal modeling, the study shows that CBCT radiomics has the potential to provide earlier insight into tumor response than conventional post-treatment imaging, thereby supporting timelier intervention strategies in liver SBRT.

#### 3.2.5. Prostate cancers

Across the three prostate cancer studies summarized in table 1, CBCT radiomics was explored as a means of characterizing treatment response and toxicity in a disease site where subtle soft-tissue changes are difficult to capture using conventional imaging. Although prostate CBCT suffers from inherent challenges, including limited soft-tissue contrast, streaking from fiducial markers, and a restricted FOV, each investigation implemented methodological safeguards to extract meaningful quantitative information. Bosetti *et al* (2020) addressed CBCT’s image-quality limitations by standardizing reconstruction settings and applying feature-stability analyses to identify histogram- and shape-based features robust enough for biochemical-relapse prediction within a risk-stratification framework. Their findings suggested that even under degraded CBCT contrast conditions, properly filtered radiomic features can reflect underlying biological aggressiveness. Delgadillo *et al* (2022) undertook a far more intensive technical approach, reconstructing daily CBCT scans from raw projection data using controlled combinations of iterative and filtered-back-projection algorithms, convolution filters, and quantization schemes to mitigate voxel-intensity inconsistency and scatter variations. They further corrected fiducial marker streak artifacts and applied IBSI-based preprocessing steps, including voxel resampling, gray level normalization, and Lloyd-Max discretization, before developing delta-radiomics models that predicted acute and subacute genitourinary toxicities as early as the first treatment week, achieving AUCs exceeding 0.8 for several endpoints. Mendes *et al* (2023) similarly emphasized the importance of harmonized preprocessing and feature robustness screening when using CBCT radiomics to differentiate favorable versus unfavorable prognoses, demonstrating that carefully curated pipelines can achieve clinically informative predictive accuracy even when relying on CBCT’s lower-quality intensity domain.

In summary, these investigations reveal a clear trajectory in which CBCT is being repurposed from a tool of geometric verification to a modality capable of capturing biologically relevant dynamics throughout prostate radiotherapy. By combining reconstruction refinement, artifact suppression, delta-based normalization, and stability-driven feature selection, researchers have shown that radiomic signatures derived from noisy CBCT images can nonetheless encode early markers of toxicity, symptom progression, or disease aggressiveness. Notably, the prostate studies highlight that intra-treatment radiomic trajectories often contain predictive value that is absent from baseline imaging alone, suggesting that the evolving tissue response, rather than pretreatment morphology, is key to identifying patients at risk for genitourinary complications or suboptimal therapeutic outcomes. As algorithmic pipelines mature and as access to raw projection data and automated contouring improves, CBCT radiomics in prostate cancer appears increasingly positioned to support adaptive workflows, toxicity-mitigation strategies, and personalized radiation treatment.

#### 3.2.6. Rectal cancers

Li *et al* (2023) developed an integrated longitudinal radiomic trend framework that leverages daily CBCT imaging to monitor and predict treatment response in locally advanced rectal cancer, as summarized in table 1. Although CBCT images for pelvic sites are susceptible to noise, scatter, motion blurring, and inconsistent voxel intensities, the authors implemented several strategies to address these limitations. Their approach included automated and manually verified registration of each daily CBCT scan to the planning CT, voxel resampling to a uniform grid, gray level normalization through Z-score standardization, and exclusion of outlier features using an absolute Z threshold. Radiomic features were extracted from multiple transformed image sets to enhance robustness, followed by a structured feature reduction pipeline consisting of univariate Mann–Whitney U tests and logistic regression to retain features with reproducible longitudinal patterns. By fitting least squares trajectories to longitudinal CBCT-derived radiomic features, the framework quantified patient-specific trends rather than relying on single time point values. Random forest modeling then enabled highly accurate discrimination between responders and non-responders during treatment, with predictive performance approaching an AUC of 0.98 in leave-one-out validation. The study illustrates how daily CBCT, despite its inherent acquisition limitations, can yield meaningful biomarkers when combined with rigorous preprocessing, longitudinal normalization, and trend-based modeling. It also reinforces the broader evolution of CBCT imaging from a setup verification tool to a quantitative source of dynamic biological information capable of supporting early intervention and adaptive radiotherapy in rectal cancer management.

### 3.3. Verification, validation, and uncertainty quantification for CBCT-based radiomics studies

Table 3 summarizes the studies that collectively explore the VVUQ of radiomics features derived from CBCT across various experimental and clinical settings for potential clinical applications regarding robustness and the challenges associated with this imaging technology. Fave *et al* (2015) and Bagher-Ebadian *et al* (2017) explored the reproducibility and interchangeability of radiomic features derived from CBCT and compare these to features derived from planning CT scans. Fave *et al* (2015) focused on the stability and reliability of these features under various experimental conditions, confirming the potential of certain features as robust biomarkers for clinical use. Meanwhile, Bagher-Ebadian *et al* (2017) studied the consistency of radiomic features between planning CT and CBCT, concluding that with proper calibration, these features were interchangeable, thus simplifying clinical workflows and potentially enhancing patient monitoring and treatment adaptation.

**Table 3.** Summary of VVUQ studies for radiomics modeling using CBCT image sets from phantoms and patients.

References	Image protocol	Image object	Institution	No. of image sets	Primary set made public	Vendor	X-ray tube voltage (kVp)	No. of radiomics features
Fave *et al* (2015)	Lung	Patient	Single	10	No	Varian	110	68
Bagher-Ebadian *et al* (2017)	HN	Patient	Single	18	No	Varian	120	165
Delgadillo *et al* (2021)	Pelvis	Patient	Single	10	No	Varian	125	42
Schmidt *et al* (2021)	Pelvis	Patient	Single	28	No	Varian	—	46
Wang *et al* (2021)	HN, Pelvis	Patient	Single	20	No	Varian	100, 125	18
Spuhler *et al* (2021)	Head, thorax, pelvis	Phantom	Single	3	No	Varian	100, 125	66, 89, 86
Palani *et al* (2021)	HN, thorax, pelvis	Phantom	Single	1	No	Varian	100, 110, 125	49
Adachi *et al* (2023)	Lung	Phantom	Multiple (n = 8)	15	No	Varian, Elekta, Siemens	125, 120, 6MV	1302
Xiao *et al* (2025)	HN	Patient	Single	114	No	Varian	—	93
Willam *et al* (2025)^a^	Head, breast, pelvis	Phantom	Single	80 s	No	Varian	100, 125, 125	107
Cvachovec *et al* (2025)^a^	Pelvis	Patient	Single	32	No	Varian	125	43

^a^ HyperSight™-CBCT imaging.

Delgadillo *et al* (2021) and Schmidt *et al* (2021) addressed the technical aspects and potentials of CBCT radiomics as a standalone modality. Delgadillo *et al* (2021) evaluated the impact of different imaging protocols on the reproducibility of radiomics features, finding significant variations that affect feature stability. The results suggested a critical need for protocol standardization to improve reliability. Schmidt *et al* (2021), through a twenty-eight-patient study, assessed CBCT radiomics as an independent imaging modality. The study confirmed the reproducibility of CBCT radiomics features across protocols in certain clinical settings. However, further investigation was essential because the quality of contours could bias the radiomic results.

Wang *et al* (2021), Spuhler *et al* (2021), and Palani *et al* (2021) further elaborated on the application of CBCT radiomics in diverse clinical environments. Wang *et al* (2021) examined the stability of CBCT-derived radiomics features across different disease sites and imaging conditions, emphasizing that despite some variability, certain features consistently maintain high reproducibility. Spuhler *et al* (2021) utilized a novel 3D printed phantom to evaluate the reproducibility of these features, highlighting substantial variability across different machines and protocols. This underscored the importance of consistent imaging procedures to ensure the reliability of radiomics features. Palani *et al* (2021) investigated CBCT radiomics’ viability as a standalone modality in medical imaging, suggesting that it could potentially match or replace certain CT radiomics applications with large amount of image data from daily treatment.

Adachi *et al* (2023) investigated how variations in imaging conditions affect the reproducibility of radiomic features by employing an anthropomorphic phantom and CBCT scans obtained from multiple vendors. Their findings highlighted the need for standardized imaging protocols to achieve consistent radiomics analyses, which is essential for incorporating radiomics into clinical workflows. Similarly, Xiao *et al* (2025) examined the relationship between CBCT image quality and the accuracy of radiomic features in nasopharyngeal carcinoma using deep learning–generated synthetic CT derived from conventional CBCT. The results indicated that synthetic CT can overcome the inherent image quality limitations of cone-beam acquisition, thereby enhancing the robustness and clinical applicability of CBCT-based radiomic biomarkers for treatment assessment and outcome prediction in radiation therapy.

Willam *et al* (2025) investigated radiomic feature robustness using HyperSight by scanning low-contrast organic phantoms under various conditions and validating findings with clinical prostate cancer data. The phantom experiments demonstrated exceptional repeatability with stability rates above 90% for re-scanning and repositioning tests. Clinical validation using 16 prostate patients revealed that while the prostate maintained 63.6% feature stability between sequential scans, critical organs like bladder and rectum showed only 15% stability due to filling variations and morphological changes during a 24-minute interval between images. Despite these challenges in mobile anatomy, they suggested that HyperSight’s enhanced image quality supports could automate radiomics workflows for monitoring tissue changes throughout fractionated radiation therapy courses.

Cvachovec *et al* (2025) evaluated the longitudinal stability of radiomic features extracted from HyperSight-CBCT imaging in 32 prostate cancer patients undergoing adaptive prostate SBRT. The researchers found that HyperSight, which provides diagnostic-grade image quality, enabled reliable radiomic feature extraction with 93.0% of features in organs at risk showing very high stability, though target structures showed lower stability (67.4%) that decreased with increasing radiation dose. The findings demonstrate that HyperSight’s superior imaging quality makes it suitable for quantitative radiomic analysis during adaptive radiotherapy, potentially enabling real-time monitoring of treatment response and establishing a framework for developing CT-based radiological biomarkers in prostate cancer radiation therapy.

This section summarized the VVUQ-related articles that emphasized the promising role of CBCT radiomics in enhancing diagnostic, prognostic, and therapeutic strategies in radiotherapy. They highlighted the critical need for rigorous validation, standardization, and consistent imaging protocols to fully harness the potential of CBCT radiomics in clinical applications, ensuring that it could be a reliable and effective tool in the healthcare setting.

#### 3.3.1. Summary of radiomic features evaluated in VVUQ studies

A summary of radiomic features that have been evaluated for repeatability and reproducibility across CBCT-based VVUQ studies is provided in table 4. These studies span lung, head and neck, and prostate clinical settings, as well as multiple phantom-based experiments designed to isolate scanner-, reconstruction-, and contour-related uncertainties. Despite substantial variability introduced by CBCT imaging physics, reconstruction algorithms, patient anatomy, and longitudinal dose effects, several consistent patterns emerge. In lung CBCT test–retest imaging, texture-based features demonstrate higher stability than first-order metrics, with a subset showing reproducibility across acquisition settings. In head and neck CBCT, Bagher-Ebadian *et al* (2017) identified broad robustness of histogram, run-length, wavelet, Gabor, and local binary pattern features to smoothing and noise, with only a small set of Haralick features exhibiting sensitivity to modality differences between pCT and CBCT. Prostate-focused VVUQ studies show that second-order features, particularly GLCM (homogeneity, correlation, dissimilarity) and GLRLM (short/long run emphasis, run percentage, gray-level emphases), are generally the most repeatable across reconstruction parameters, deformable contour propagation, and short-term test–retest scenarios. Phantom experiments further confirm that many texture features maintain high reproducibility across scanner types and institutions, especially when filtered (Laplacian of Gaussian or wavelet-based) features are used. Newer imaging platforms such as Hypersight CBCT demonstrate markedly improved stability across both phantom and patient datasets, with longitudinal analyses showing that second-order features remain the most robust, while first-order and shape features are more sensitive to anatomic and dose-related changes during treatment. Together, these results highlight a consistently reproducible subset of radiomic features suitable for CBCT-based biomarker development and longitudinal delta-radiomics analysis in adaptive radiotherapy workflows.

**Table 4.** Summary of radiomic features evaluated for repeatability and reproducibility across CBCT-based VVUQ studies.

References	Validated repeatable and reproducible Features	Note
Fave *et al* (2015)	**Texture:** 37 features with CCC > 0.9 and low volume dependence; subset of 4–13 features reproducible across CBCT systems when imaging protocols are consistent	Lung CBCT test–retest images used to compute feature CCC and assess volume dependence; robust features further evaluated in texture and motion phantoms to test sensitivity to scanner, scatter, and motion
Bagher-Ebadian *et al* (2017)	**IBHF:** all histogram features except Skewness and Kurtosis, **GLRL:** SRE, LRE, GLN, RLN, RP, LGRE, HGRE, **LAWS:** nine energy and most entropy features, **LBP:** six local binary pattern statistics, **DOST:** segment energy and entropy features, **2DWT:** energy and entropy maps from multilevel wavelet decomposition, **2D Gabor:** energy and entropy maps across scales and orientations, **GLCM:** 17 Haralick features excluding Contrast, Dissimilarity, Difference variance	Most features robust to low-power Gaussian noise (SNR ≥ 50) and low-pass smoothing, whereas high-pass LoG filtering produces large mean absolute percent changes. Only 15 of 165 features show significant differences between baseline pCT and CBCT, supporting modality interchangeability for most radiomics features.
Delgadillo *et al* (2021)	**GLSZM:** Zone Percentage (ZP), **NGTDM:** Coarseness, Strength, **GLRLM:** Long Run Emphasis (LRE), Low Gray-Level Run Emphasis (LGRE)	Repeatability assessed using CCC; reproducibility defined by pCT–CBCT correlation. These features also reproducible in ~10% of settings.
Schmidt *et al* (2021)	**GLCM:** Contrast, Correlation, Dissimilarity, Energy, Entropy, **GLRLM:** SRE, LRE, LGRE, HGRE, RLN, SRHGLE, LRHGLE, RP, **GLSZM:** LGZE, HGZE, ZSN	Shows that prostate CBCT radiomics is largely robust to DIR contouring variability.
Wang *et al* (2021)	**First-order:** Skewness, Entropy, **GLCM:** Energy, Joint Entropy, Dissimilarity, Correlation, **GLRLM:** SRE, LRE, RLN, HGLRE, SRHGLE, LRHGLE, RP	GLCM-Energy is least stable across time. Most features degrade after 5 days (treatment-induced variation).
Spuhler *et al* (2021)	**GLCM:** multiple stable texture features, **GLRLM:** multiple stable run-length features, **NGTDM:** multiple stable neighbourhood texture features	Inter-LINAC reproducible features remained numerous (61–65). Phantom-based confirmation of protocol stability.
Palani *et al* (2021)	**First-order:** intensity-based features, **GLCM:** multiple co-occurrence features, **GLRLM:** multiple run-length features	ICC significant versus CT for intensity, GLCM and GLRLM features; demonstrates that CBCT radiomics can approximate CT when noise is controlled
Adachi *et al* (2023)	**LoG-GLRLM:** multiple run-length features, **Wavelet-GLDM:** dependence features, **Wavelet-GLCM:** energy and entropy features	Shows that filtered features (LoG/wavelet) are most stable under scanner variability.
Xiao *et al* (2025)	GLCM, GLRLM, GLSZM, NGTDM, GLDM	DL enhancement increases radiomic similarity to CT ground-truth.
Willam *et al* (2025)^a^	**Shape:** most shape features, **First-order:** most first-order features, **Second-order (GLCM, GLRLM, GLSZM, NGTDM, GLDM):** Homogeneity, SRE, LRE, LRHGE, RP	Phantom re-test shows 98%–100% of features stable (CCC > 0.90); clinical prostate test–retest shows 63% stable features; HyperSight CBCT substantially improves radiomic feature stability
Cvachovec *et al* (2025)^a^	**GLRLM:** LRHGE, LRE, RP, SRE, **GLCM:** Homogeneity, Dissimilarity, **First-order:** HU-std, **GLSZM:** SZE, SZLGE, ZLNU, **Shape:** Sphericity	Second-order features outperform first-order and shape features; prostate radiomic features exhibit fraction- and dose-dependent variability

*Abbreviations:* IBHF: intensity-based histogram features; GLRL: gray-level run length; LAWS: law’s textural information; DOST: discrete orthonormal stockwell transform; 2DWT: two-dimensional wavelet transform; LBP: local binary pattern; GLCM: gray level co-occurrence matrix; GLSZM = gray-level size zone matrix; NGTDM = neighborhood gray-tone difference matrix; GLRLM = gray-level-run length matrix; GLDM = gray-level dependence matrix; CCC = concordance correlation coefficient; ICC = intra-class correlation coefficients; LoG: Laplacian of Gaussian.

^a^ HyperSight™-CBCT imaging.

### 3.4. 4D-CBCT enhanced radiomics feature studies

Table 5 provides an overview of two studies by Zhang *et al* (2021, 2022) that focused on improving the image quality of 4D-CBCT for robust radiomic analysis through deep learning (DL) approaches. In their 2021 work, the authors demonstrated that the quality of 4D-CBCT images has a substantial impact on the extraction and precision of radiomic features. They further showed that the DL model TecoGAN markedly enhanced image quality and feature accuracy, particularly in cases involving larger tumors. Their consecutive study (Zhang *et al* 2022) in 2022 advanced this by developing a patient-specific DL model, which more effectively corrected radiomic feature errors compared to universal models, providing a tailored approach that enhanced the precision of radiomics in predicting radiotherapy outcomes. Collectively, these studies highlighted the potential of DL to improve the reliability and specificity of radiomic analyses in cancer treatment.

**Table 5.** Summary of VVUQ studies for CBCT-based radiomics using simulated 4D-CBCT.

References	Image protocol	Image source	No. of image sets	Primary set made public	No. of radiomics features	Model	Objective
Zhang *et al* (2021)	Lung	Patient	3	Yes	540	TecoGAN	Uncertainty quantification
Zhang *et al* (2022)	Lung	Patient	4	Yes	946	Pix2PixGAN	Quality enhancement

*Abbreviations:* GAN = generative adversarial network.

## 4. Discussion

We followed the PRISMA protocol to systematically review CBCT-only radiomics for EBRT. The integration of CBCT-based radiomics into the field of EBRT can potentially induce a paradigm shift in cancer radiotherapy, offering more personalized and precise therapeutic strategies. CBCT’s ability to provide in-room, high-resolution, volumetric imaging has made it a valuable tool in guiding daily radiotherapy. When combined with radiomics, which extracts and analyzes large sets of quantitative features from medical images, CBCT offers a promising approach to enhance tumor characterization, adaptive treatment planning, and monitoring of treatment responses.

Figure 2 depicts the growing interest in developing predictive models using CBCT-based radiomics across a variety of cancer types, including HNSCC, NSCLC, ESCC, and prostate and rectal cancers. Table 1 summarizes how these studies have employed various machine learning models to predict outcomes like tumor response, patient survival, and tissue toxicity. Delta-radiomics, which tracks temporal changes in radiomic features (Iliadou *et al* 2022, Yang *et al* 2024), has been demonstrated to offer important insights into early treatment responses and inform adaptive radiotherapy approaches. By allowing treatment plans to be tailored to each patient’s response, this strategy holds promise for enhancing clinical outcomes.

**Figure 4.**
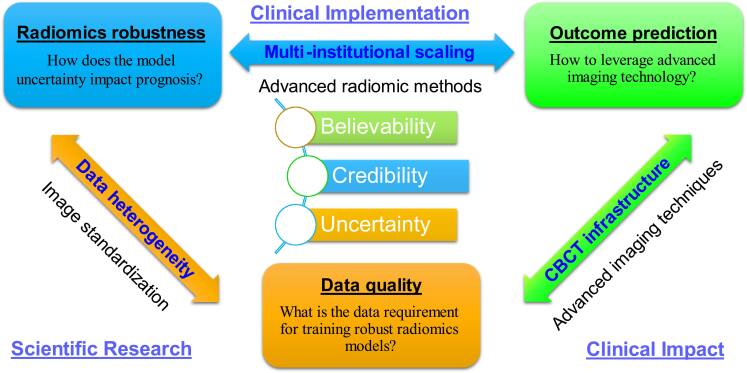
Overview of challenges for implementing robust CBCT-based radiomics for multi-institutional cancer prognosis, including potential online adaptive treatment based on early tumor response and normal tissue toxicity.

However, implementing CBCT-based radiomics in clinical practice presents multiple challenges. Figure 4 provides an overview of these challenges, which we have categorized into three main areas: data quality, radiomics robustness, and cancer prognosis. Each category is associated with a specific question, as outlined below.

• What is the data requirement for training robust radiomics models?
• How does the model uncertainty impact prognosis?
• How to leverage advanced imaging technology?

**Figure 5.**
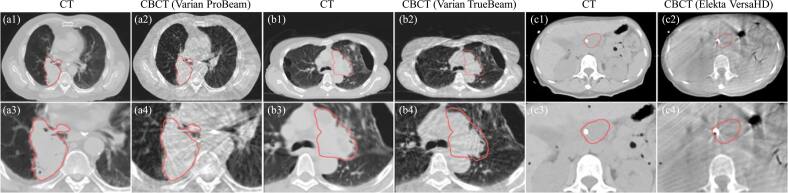
Comparisons of image quality between CT and CBCT acquired from Varian and Elekta systems. (a1)–(a2) show images from CT and CBCT on the Varian ProBeam system (125 kVp), (b1)–(b2) show images from CT and CBCT on the Varian TrueBeam system (125 kVp), and (c1)–(c2) show images from CT and CBCT on the Elekta VersaHD system (120 kVp). (a3)–(a4), (b3)–(b4), and (c3)–(c4) present zoomed-in views of regions near the tumor volume, highlighted with red contours in (a1)–(a2), (b1)–(b2), and (c1)–(c2). All figures were prepared de novo for this review using institutional data.

For the first data requirement question, we break it down further into data quantity and data uncertainty. Many studies in table 2 highlight limitations in data quantity due to a lack of multi-institutional testing. The Health Insurance Portability and Accountability Act (U S Department of Health and Human Services 2024) strictly protects medical image data, creating obstacles for collaboration across institutions. Generative artificial intelligence offers a potential solution by utilizing large language models (Liu *et al* 2023) for semantic reasoning, which can aid in de-identifying protected healthcare information and facilitate multi-institutional data sharing. Data uncertainty can arise from imaging artifacts, which compromise image quality and introduce uncertainties in radiomic analysis. Figure 5 depicts the comparisons between CT and CBCT from Varian and Elekta systems, and the CBCT images show various degrees of artifacts, which can bias the information extracted from the target volumes. These artifacts cause inconsistencies in feature extraction, impacting the accuracy and reliability of predictive models. Furthermore, table 2 indicates that variability in imaging protocols and equipment across different institutions adds complexity, making it challenging to standardize radiomic features and compare results across studies. This issue of data heterogeneity underscores the need for standardized medical imaging (Selim *et al* 2020, 2023) to ensure CBCT-based radiomic models with the capability of reproducibility and generalizability.

The second question addresses the robustness of radiomics models. Several studies (Aerts *et al* 2014, Parmar *et al* 2014, Zwanenburg *et al* 2020) have shown that different initial feature sets can significantly impact the predictions of these models. The selected features determine the information available to the model for making predictions. If key features are excluded, the model may miss crucial patterns in the data, leading to lower accuracy. Conversely, incorporating an excessive number of features, particularly those that are irrelevant or redundant, may lead the model to overfit the training data, reducing its ability to generalize to new, unseen datasets. Highly correlated features can provide redundant information and including them without proper handling can skew predictions and increase model complexity unnecessarily. A model with a well-chosen, smaller feature set is generally easier to interpret, which is crucial in clinical settings where understanding the basis of a prediction is important for trust and decision-making. Additionally, larger feature sets increase the computational complexity of model training and prediction. A model trained on a smaller, well-curated feature set may generalize better to new data, enhancing its robustness and reliability. Therefore, a standardized commissioning framework can potentially enable the deployment of radiomics models across different institutions, ensuring that an available, relevant, and adequately evaluated feature set is used for building effective radiomics models.

**Figure 6.**
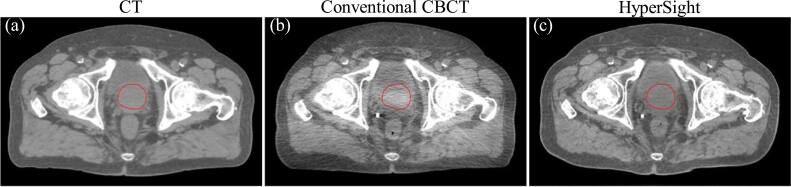
Comparisons of image quality between CT and CBCT acquired from Varian TrueBeam with conventional CBCT and HyperSight systems. (a), (b) show images from prostate treatment. The prostate is highlighted with red contours. The CBCT images for each treatment site were acquired from the same patient but different treatment fractions. All figures were prepared de novo for this review using institutional data.

The final question concerns the availability of advanced CBCT techniques. Robar *et al* (2024) and Kim *et al* (2024) evaluated Varian’s newly developed kV/CBCT imaging system, HyperSight, and found that this innovative CBCT platform significantly reduced image artifacts and improved uniformity compared to conventional LINAC-mounted CBCT. HyperSight (Cvachovec *et al* 2025, Willam *et al* 2025) also demonstrated considerable enhancements in contrast-to-noise ratio, noise reduction, and CT number calibration accuracy, closely aligning with the performance of a CT scanner (figure 6). This new imaging infrastructure has the potential to enhance clinical efficiency and reduce the inherent uncertainty of radiomics models by providing high-quality image data. As illustrated in figure 4, data quality and model robustness significantly influence the uncertainty of predictive capability and the credibility of CBCT-based radiomics methods. While CBCT exposes a significant tissue volume to radiation (Ding *et al* 2018), requiring a careful equilibrium between clinical benefits and patient safety, a thorough investigation into the imaging dose specific to HyperSight has yet to be conducted. Robar *et al* (2024) showed that HyperSight had statistically inferior uniformity compared to CT. They also found that free-breathing HyperSight acquisitions remained susceptible to motion artifacts, which compromised the image usability. To address these limitations, a strategic integration of HyperSight with AI-based synthetic CT techniques (Pan *et al* 2024, Chang *et al* 2024, Chen *et al* 2024, Peng *et al* 2024a, 2024b, Chen *et al* 2025, Pan *et al* 2023) and rigorously validated radiomics models could be pursued. This synergy may establish the credibility of CBCT-based radiomic analyses, paving the way for enhanced adaptive radiotherapy workflows and a paradigm shift in precision radiation oncology.

Several works listed in table 1 employ delta-radiomics approaches to capture treatment-induced changes over time, a methodology that has demonstrated notable advantages for toxicity prediction in radiotherapy. A systematic review and meta-analysis by Tan *et al* (2023) synthesized evidence from 13 studies across various malignancies including head and neck, NSCLC, nasopharyngeal, oesophageal, oropharyngeal, and prostate cancers, and found that delta-radiomics models achieved a pooled AUC of 0.80 (95% CI: 0.69–0.90), outperforming non-delta radiomics models (AUC: 0.78). The principal advantage of delta-radiomics lies in its capacity to characterize longitudinal variations in imaging features across multiple acquisition time points, thereby capturing patient-specific and organ-specific heterogeneity as well as individual sensitivity to radiation-induced damage. Notably, the best-performing model in the review attained an AUC of 0.90 for late xerostomia prediction by incorporating morphological features such as parotid gland surface changes. Regarding the optimal timing for CBCT-based radiomics analysis, evidence from the reviewed studies suggests that early-to-mid treatment time points may be particularly informative for toxicity prediction. Specifically, van Dijk *et al* (2019) reported that parotid gland surface changes were most predictive at week 3 of treatment, while Rosen *et al* (2018) found that mean Hounsfield unit intensity changes at weeks 3–4 and volume changes at weeks 5–6 were significant predictors of xerostomia severity. Similarly, Alam *et al* (2021) demonstrated that oesophageal volume changes between weeks 1 and 2 achieved an AUC of 0.80 for predicting acute oesophagitis. These findings suggest that critical morphological and textural changes occur within the first few weeks of treatment, offering a potential window for early intervention or treatment adaptation. When comparing pre-treatment planning CT to intra-treatment CBCT-based predictions, several studies demonstrated complementary value. Pre-treatment CT provides baseline anatomical and textural characteristics with superior image quality, while serial CBCT acquisitions capture dynamic treatment-induced changes despite inherent image quality limitations. The combination of baseline features with delta-radiomics features derived from CBCT has shown improved predictive performance compared to either approach alone. For example, van Dijk *et al* (2017) reported that integrating acute xerostomia scores with delta image biomarker features improved the AUC from 0.85 to 0.90.

However, delta-radiomics approaches face several limitations that warrant consideration. The meta-analysis revealed substantially higher heterogeneity among delta-radiomics models (I^2^ = 73%) compared to non-delta models (I^2^ = 27%), attributable to variabilities in longitudinal datasets, inconsistent acquisition parameters, and morphological changes in the region of interest across time points. Additionally, the lack of standardization in CBCT imaging protocols, including differences in image resolution, slice thickness, and reconstruction algorithms, poses challenges for feature reproducibility and multi-institutional validation. Given that CBCT is increasingly utilized for longitudinal imaging in adaptive radiotherapy, ensuring the reliability and reproducibility of CBCT-derived radiomic features becomes paramount. Therefore, another central focus of this review is the evaluation of verification, validation, and uncertainty quantification (VVUQ) in CBCT-based radiomics studies (table 3). The studies highlighted the significance of examining the reproducibility of radiomic features both within a single institution and across multiple centers. In several studies, the use of phantoms offered critical insights into how radiomic features vary across different imaging systems and acquisition protocols. Despite the challenges, the consistent results in some respects suggest that CBCT radiomics has the potential to serve as a robust imaging modality, particularly in the context of oncology for treatment planning and monitoring. To fully harness this potential, there is a pressing need for the development of standardized procedures and guidelines for CBCT image acquisition, feature extraction, and model validation.

The use of 4D-CBCT in radiomics is an emerging area that shows great promise in enhancing image quality and improving the accuracy of radiomic analyses. As shown in table 5, studies employing DL models, such as GANs, have demonstrated the ability to improve 4D-CBCT image quality, thereby reducing uncertainty in radiomic feature extraction. This approach is particularly beneficial in cases where tumor motion is a concern, such as in lung and liver cancers. These advances in 4D-CBCT image enhancement reflect the broader advantages of DL-based radiomics described by Abdollahi *et al* (2022). Unlike traditional hand-crafted features, DL-based radiomic features are not pre-defined but rather generated automatically by neural networks such as recurrent neural networks, convolutional neural networks, and GANs. This allows features to be extracted from both segmented and non-segmented images for end-to-end clinical applications. DL has been successfully integrated into multiple radiomics pipeline steps, including automated tumor segmentation, outcome prediction, and treatment response assessment, all of which are directly relevant to improving 4D-CBCT-based radiotherapy workflows. Furthermore, studies cited in (Abdollahi *et al* 2022) demonstrate that combining DL with radiomics improves predictive accuracy for epidermal growth factor receptor mutation status, tumor grading, staging, and survival outcomes across multiple cancer types. The authors envision ‘next-generation AI-radiomics-guided radiation therapy’ where DL networks integrated with advanced imaging modalities, including CBCT, CT, MRI, MR-Linacs, and PET-based delivery systems, will enable truly individualized radiotherapy. This individualized approach would span from patient selection and automated target delineation to adaptive treatment and post-therapy response assessment. However, the review also acknowledges DL-specific challenges, including the need for larger training datasets, optimal architecture selection, and improved network interpretability before widespread clinical translation can occur. By applying these DL methodologies to 4D-CBCT radiomics specifically, there is significant potential to improve the reliability and specificity of radiomic features in the presence of respiratory motion, ultimately providing more precise predictions of radiotherapy outcomes and advancing the field of personalized cancer treatment.

The integration of CBCT-based radiomics into radiotherapy holds significant potential for improving the personalization and effectiveness of cancer treatment. While the field is still in its early stages, the advancements in method development and the increasing focus on VVUQ suggest a positive trajectory towards clinical implementation. Addressing the challenges related to image quality, standardization, and validation is essential for realizing the full potential of this technology. Future research should prioritize multi-institutional collaborations and the development of standardized imaging protocols to ensure the reproducibility and clinical applicability of CBCT-based radiomic models. As these challenges are overcome, CBCT-based radiomics is poised to play a crucial role in the advancement of personalized radiotherapy, ultimately improving patient outcomes.

## 5. Concluding remarks

The integration of CBCT-based radiomics in radiotherapy represents a promising frontier for enhancing personalized treatment approaches. Despite the challenges posed by imaging artifacts and variability in imaging protocols, ongoing research and methodological improvements are driving the field towards more accurate and individualized cancer treatment. The adoption of multi-institutional collaborations and standardization of imaging protocols will be crucial in realizing the full potential of CBCT-based radiomics in clinical practice. New technologies in terms of both hardware and software that improve CBCT image quality, utility, and dosimetry will make CBCT a more valuable resource moving forward.

## Data Availability

The data cannot be made publicly available upon publication because they contain sensitive personal information. The data that support the findings of this study are available upon reasonable request from the authors.
